# Neural Competition for Conscious Representation across Time: An fMRI Study

**DOI:** 10.1371/journal.pone.0010556

**Published:** 2010-05-10

**Authors:** Heleen A. Slagter, Tom Johnstone, Iseult A. M. Beets, Richard J. Davidson

**Affiliations:** 1 Cognitive Neuroscience Group, Department of Psychology, University of Amsterdam, Amsterdam, The Netherlands; 2 Waisman Laboratory for Brain Imaging and Behavior, University of Wisconsin, Madison, Wisconsin, United States of America; 3 Centre for Integrative Neuroscience and Neurodynamics, School of Psychology and CLS, University of Reading, Reading, United Kingdom; 4 Department of Psychology, University of Wisconsin, Madison, Wisconsin, United States of America; Macquarie University, Australia

## Abstract

**Background:**

The information processing capacity of the human mind is limited, as is evidenced by the attentional blink (AB) - a deficit in identifying the second of two temporally-close targets (T1 and T2) embedded in a rapid stream of distracters. Theories of the AB generally agree that it results from competition between stimuli for conscious representation. However, they disagree in the specific mechanisms, in particular about how attentional processing of T1 determines the AB to T2.

**Methodology/Principal Findings:**

The present study used the high spatial resolution of functional magnetic resonance imaging (fMRI) to examine the neural mechanisms underlying the AB. Our research approach was to design T1 and T2 stimuli that activate distinguishable brain areas involved in visual categorization and representation. ROI and functional connectivity analyses were then used to examine how attentional processing of T1, as indexed by activity in the T1 representation area, affected T2 processing. Our main finding was that attentional processing of T1 at the level of the visual cortex predicted T2 detection rates Those individuals who activated the T1 encoding area more strongly in blink versus no-blink trials generally detected T2 on a lower percentage of trials. The coupling of activity between T1 and T2 representation areas did not vary as a function of conscious T2 perception.

**Conclusions/Significance:**

These data are consistent with the notion that the AB is related to attentional demands of T1 for selection, and indicate that these demands are reflected at the level of visual cortex. They also highlight the importance of individual differences in attentional settings in explaining AB task performance.

## Introduction

At any given moment, multiple representations compete for limited attentional resources and for control of behavior. Many views of attention posit the existence of top-down signals that play a critical role in resolving this competition by selectively enhancing the representations that underlie our conscious perceptions, while inhibiting irrelevant information (e.g., [1], [2], [3]). Confirming this idea, single-cell recordings in animals and human neuroimaging studies have shown that attention not only facilitates the processing of attended information, by enhancing activity of sensory brain areas that represent this information, but also selects behaviorally relevant stimuli from among distracters, by inhibiting responses to distracter information (for review see e.g., [4]).

Competition for attentional resources not only occurs when stimuli are presented simultaneously, but also when they are presented separately in close temporal proximity, as is illustrated by the attentional blink (AB) deficit [5]. This deficit occurs when subjects have to detect two target stimuli (T1 and T2) embedded in a rapid stream of distracter events. When T2 is presented within 200 to 500 ms of T1, it is often not detected. Cognitive accounts of this target processing deficit generally agree that it results from competition between stimuli for conscious representation (e.g., [6], [7], [8], [9], [10], [11], [12], [13]). Many of the available accounts also share the assumption that processing T1 leads to the occupation of some attentional mechanism that is unavailable for processing T2 until T1 processing is completed, and thus, that the AB is related to some central bottleneck in information processing (for recent reviews, see [14], [15]). For example, two-stage theories postulate that stimuli compete for entry to a limited-capacity serial processing stage that is necessary for the stimuli to reach awareness or to elicit a response (e.g., [6], [7]). The attentional blink occurs when this stage is still engaged in T1 processing when T2 is presented. Thus, two-stage or bottleneck models propose that T1 and T2 are processed serially, and propose that the duration of T1 processing determines the AB, and hence, that the AB arises from a central bottleneck in information processing. Other models of the AB have postulated different mechanisms to explain the AB. For example, according to the resource sharing account of the AB, the AB does not reflect an immutable, structural processing bottleneck, but a processing strategy [11]. In this model, T1 and T2 are processed in parallel and are in direct competition for shared, limited resources. According to this model, the amount of attention devoted to T1 processing varies from trial-to-trial and there is a reciprocal relationship between the amount of attention devoted to T1 and T2 processing: The more attentional resources T1 demands, the fewer are available for T2. While bottleneck models also predict that T1 processing influences attentional processing of T2, since the two targets are assumed to be processed serially, T1 processing determines resource availability for T2 rather in an all-or-none fashion: While T1 is being processed, no resources are available for T2 processing, while in principle, once T1 processing has finished, all resources should be available again for T2. In another recently proposed model of the AB, T1 and T2 are also processed in parallel, as in the resource sharing account, but the AB is not due to T1 processing per se, but caused by the distracter immediately following T1 [15]. In this model, working memory employs an input filter that enhances stimuli that match the target set, and inhibits non-target stimuli: When T1 is presented, it elicits an attentional boost, but because of its temporal proximity to T1, the first item after T1 is also boosted. When this is a distracter, the input filter will subsequently issue an inhibitory signal. This inhibitory signal will then transiently suppress subsequently presented stimuli, including T2. This inhibitory signal is thus the cause for the AB, according to the so-called boost-and-bounce theory. Notably, in this model, the strength of the attentional response elicited by T1 affects the strength of the suppressive response triggered by the distracter immediately following T1. Therefore, although the AB is not due to T1 processing per se in the boost-and-bounce theory, attentional processing of T1 also influences whether or not an AB to T2 will occur. Thus, the available accounts disagree in the specific mechanisms underlying the AB, in particular about how attentional processing of T1 influences T2 processing.

Although the different models of the AB are architecturally quite different, they make largely indistinguishable behavioral predictions [16]. For example, studies of T1 difficulty often show that greater “difficulty” or T1 processing time leads to a larger AB (e.g., [9], [17], [18], [19]). This is generally consistent with either a longer bottleneck or a greater proportion of resources devoted to T1 at the expense of T2. Neuroimaging methods–by revealing the neural mechanisms underlying the AB–may therefore provide additional important information that may help distinguish between the available accounts of the AB. In line with prior behavioral studies (e.g., [9], [17], [18], [19]) and in favor of the general idea that the amount of attentional resources devoted to T1 influences the likelihood that T2 is detected, several recent event-related potential (ERP) studies [11], [20], [21], [22], [23] have reported a relationship between the amplitude of the T1-elicited P3b, a brain-potential index of resource allocation [24], and conscious T2 perception. Some of this work has also observed a reciprocal relationship between the amount of attentional resources devoted to T1 and T2 processing, in line with the resource-sharing account of the AB [11]. For example, Kranczioch et al. [20] found that a bigger T1-elicited P3b was associated with a smaller T2-elicited P3b, suggesting that a greater allocation of resources to T1 reduced the amount of resources that can be allocated to T2. This reciprocal relationship can not easily be explained by two-stage theories of the AB, in which T2 should in principle have access to all resources once T1 processing has finished. However, other ERP studies have failed to find a reciprocal relationship between the amplitudes of the T1- and T2-elicited P3b's. For example, Slagter et al. [25], [26] reported a mental training-related decrease in the amplitude of the T1-elicited P3b, which was associated with an increase in T2 detection rates. Yet, this decrease in T1-elicited P3b amplitude was not accompanied by a corresponding increase in the amplitude of the P3b to detected T2's, arguing against a reciprocal relationship between the amount of resources devoted to the two targets. Of further importance, it has been argued that the P3b does not reflect resource allocation in the AB paradigm, but rather bottom-up target strength [27]. Thus, ERP studies have shown differences in neural processing of T1 as a function of conscious T2 perception, but are inconclusive as to how T1 encoding influences T2 processing.

While the temporal resolution of ERP is high, it is not accompanied by the high spatial sensitivity of functional magnetic resonance imaging (fMRI). Previous fMRI studies have shown greater activity in a network of frontal and parietal brain areas in no-blink vs. blink trials, suggesting a role for this network in conscious target perception [28], [29], [30], [31], [32]. In line with the large body of literature showing that top-down attention modulates activity in stimulus-specific visual areas (e.g., [4], [33]), several fMRI studies have also reported T2 detection-related differences in activity in temporal and occipital brain areas [28], [31], [32], [34], [35], [36]. These latter observations indicate that how much attention can be allocated to T2 significantly modulates activity in lower-level brain areas that represent T2. Although previous fMRI studies have provided valuable insights into the network of brain areas involved in conscious T2 detection, due to the low temporal resolution of the fMRI technique, the design of these studies did not permit examination of how differences in attentional processing of T1 might affect T2 processing.

The present study used the high spatial resolution of fMRI to examine at the neural level how attentional processing of T1 may influence conscious T2 perception. Our research approach was to design T1 and T2 stimuli that activate distinguishable brain regions involved in visual categorization and representation, and then to measure activity in these target-object representation brain regions as a function of conscious T2 perception. This approach allowed us (i) to examine whether T1 processing at the level of object-representation is predictive of T2 detection, (ii) to test whether T1 and T2 directly compete for shared attentional resources, as the resource-sharing account of the AB [11] postulates.

Regardless of whether T1 and T2 directly compete for shared attentional resources, it is clear that some aspect of having to encode a first target impairs the detection of a subsequently presented second target. Yet, since the design of previous fMRI studies did not permit dissociation of T1 and T2 processing, it is unclear how at the neural level, T1 encoding may influence T2 processing. Based on a large body of literature showing that top-down signals selectively enhance the representations that underlie our conscious perceptions (e.g., [1], [2], [3]), if attentional processing of T1 affects T2 processing, one may expect to find 1) greater activation in T1-object representation areas in blink versus no-blink trials, and 2) activation in T1-object representation areas to predict individual differences in AB size. We tested these predictions using region of interest (ROI) analyses.

Second, to determine whether or not T1 and T2 are in direct competition for shared attentional resources, as the resource sharing account of the AB predicts [11], we examined whether activity in the T1- and T2-object representation areas co-varied from trial-to-trial as a function of whether T2 was seen (no-blink trial) or missed (blink trial) using a context-dependent functional connectivity analysis (or psychophysiological interaction (PPI) analysis; [37], [38]). If there is a reciprocal relationship between the amount of attentional resources devoted to T1 and T2 processing, one would expect activity in these areas to co-vary as a function of behavior.

## Methods

### Behavioral pilot experiment

#### Subjects

Sixteen healthy, right-handed subjects (ten females, age range: 18–29 years, mean age: 21.1 years, SD age: 3.1 years) participated in a behavioral pilot study, which was conducted first to establish that a robust AB can be obtained with our AB task paradigm (see below). The subjects were recruited via the use of flyers posted on campus and in a large number of public places (shops, libraries, etc.) in Madison, WI. They gave written informed consent, and were paid $10 per hour for their participation. The study was approved by the research ethics committee of the University of Wisconsin.

#### Single and dual tasks

**Figure 1:**
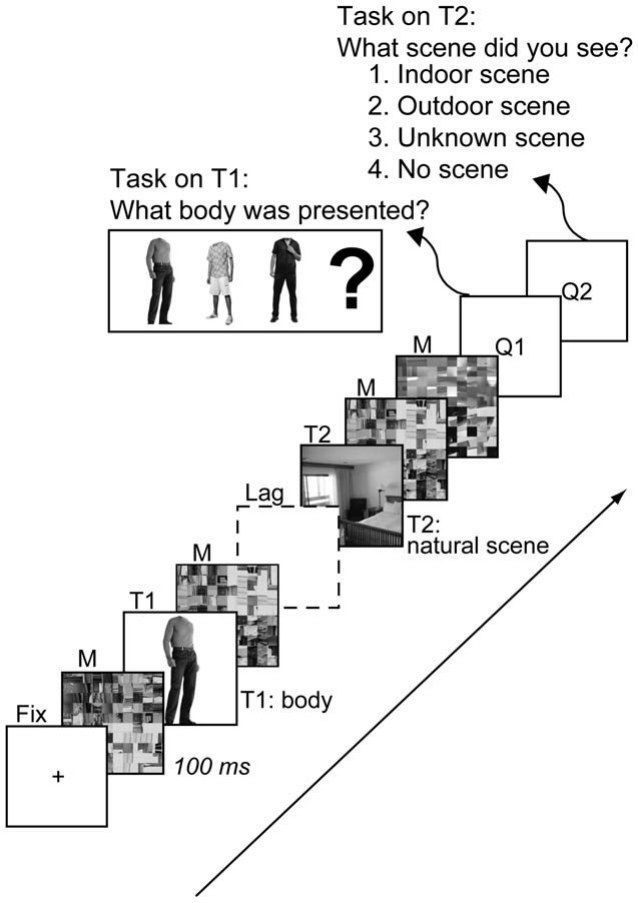
The attentional blink task. **Example of a trial.**

Subjects performed two tasks: 1) an AB (or dual) task (detect T1 and T2), and 2) a single task (detect only T2). In the dual task (Figure 1), each trial started with a task preparation period of variable duration (jittered between 2000 and 8000 ms with steps of 100 ms; average 5000 ms) during which a central fixation cross was shown. The color of this fixation cross turned from black to green 1800 ms before the onset of a rapid serial stream of grayscale images (12.8°×12.8°; presented for 100 ms each with no inter-stimulus interval), orienting subjects to the upcoming task. Subjects searched the stream for two target images: a body without a head (T1) and a natural scene (T2). T1 was randomly drawn from a set of 10 (headless) body stimuli (from [39]), displayed on grey background. T2 could follow T1 after 200, 400, 600 or 800 ms, with equal probability, and was randomly drawn from a set of 54 indoor and 54 outdoor scenes (from [31]), with equal probability of indoor and outdoor scene presentation. The T1 and T2 stimuli were chosen as they activate selective regions of the visual cortex, namely the extrastriate body area (EBA) and the parahippocampal place area (PPA). The EBA shows selectivity for bodies, while the PPA shows selectivity for natural scenes [39], [40]. T2 was always shown at the second-to-last position in the image stream. In 20% of trials, T2 was replaced by a scrambled version of a scene image (T2-absent trials). The distracter images were also scrambled versions of the scene images and were randomly drawn (without replacement) from the pool of 108 images. The scrambled images were created by dividing the image into 100 squares and randomly scrambling their positions. Thin black grids were drawn over the scrambled (and intact) images to occlude the boundaries of blocks (cf. [31]). The image stream was followed by a fixation period of variable duration (jittered between 2000 and 6000 ms with steps of 100 ms; average 4000 ms). A trial ended with the presentation of T1 response and T2 response displays, each for 1800 ms. During the T1 response displays, three images of headless bodies were shown (one of which was T1), as well as an image of a question mark (see Figure 1). Subjects decided by key press which of the headless body images was T1 or indicated that they had not seen T1 by selecting the question mark image. During the T2 response displays, they selected whether an indoor scene, an outdoor scene, an unknown scene, or no scene had been presented. The “unknown scene” response option was included in case subjects perceived the layout of a scene but were not certain whether it was indoor or outdoor (cf. [31]). Stimuli were presented on a gray (40 cd/m2) background at the center of a computer screen using E-Prime software (Psychology Software Tools, Pittsburgh, PA) with a screen resolution of 800 by 600 pixels. Subjects were instructed to emphasize the first target task over the second target task.

The single task was identical in design to the dual task, except that subjects were instructed to search for the scene target only and to ignore the body stimulus. Subjects first practiced the tasks for 30 trials each and then performed two runs of 50 trials each of both tasks in counterbalanced order.

### fMRI experiment

#### Subjects

Twenty-four right-handed subjects (nineteen females, age range: 18–28 years, mean age: 21.3 years, SD age: 2.4 years) participated in both experimental sessions (see below). They all met standard MRI compatibility criteria and had never been diagnosed with a psychiatric or neurological disorder. The subjects were recruited via the use of flyers posted on campus and in a large number of public places (shops, libraries, etc.) in Madison, WI. They gave written informed consent, and were paid $100 for their participation. The study was approved by the research ethics committee of the University of Wisconsin.

#### Procedure

The subjects participated in two sessions. In the first session, they were familiarized with an MR environment and practiced the AB (i.e., dual) task in a mock scanner. Performance in this session was used to determine whether or not a subject would be invited back for the neuroimaging session. This was done to ensure that each subject had enough blink and no-blink trials to be included in our critical analysis comparing neural activity in blink vs. no-blink trials (see below). Specifically, only subjects who were able to detect both targets on at least 30% of short-interval trials, but no more than 70% of short-interval trials, were invited back for the second MRI session. In this session, they first performed a one-back detection localizer task, followed by the AB task.

#### Session 1

After a detailed explanation of the study procedures, subjects first practiced the AB task (Figure 1) for 16 trials sitting behind a desk. Following this initial practice block, they performed two runs of 26 trials each of the AB task in a mock scanner, while listening to simulated scanner sounds via earbud headphones. The AB task was identical in design to the dual task used in the behavioral pilot experiment with the exception that T2 was present on 77% of trials, and when present, followed T1 after either 400 ms (77% of T2-present trials) or 800 ms (23% of T2-present trials). Stimuli were presented via a fiber-optic goggle system (Avotec, Stuart, FL).

#### Session 2

During their second visit, subjects first performed a one-back repetition detection localizer task (6 minutes) in the MRI scanner. This task consisted of eight alternating blocks of headless bodies and scenes (i.e., the T1 and T2 stimuli used in the AB task), with each block containing 20 bodies or scenes presented at the fixation point for 800 ms each followed by a 200 ms blank screen. Twenty-second fixation periods were interspersed with these stimulus blocks. The task also began and ended with a 20-second fixation period. Subjects searched for bodies and scenes that appeared twice in a row, and pressed a button upon repetition detection. Data collected during this task was used to localize the extrastriate body area (EBA) and parahippocampal place area (PPA) in each subject. The EBA and PPA are involved in processing body parts and natural scenes, respectively [31], [39], [40]. The localizer task was followed by three runs of 26 trials each of the AB task (see Figure 1). The task was identical to the AB task used in session 1, with the exception that the stream always contained both targets. T2 followed T1 after a short interval in 77% of trials (i.e., 60 trials), and after a long interval in 33% of trials (i.e., 18 trials). As the critical fMRI analysis focused on short-interval blink and no-blink trials, more short- than long-interval trials were included in the design to enhance statistical power. Based on power analyses as described by [41], the numbers of trials included in our fMRI analyses, albeit somewhat low, are sufficient for reliable BOLD estimates. Indeed, addition of more trials is unlikely to increase power since with this many trials the overall variance is dominated by between-subjects variance. This is exemplified by the highly significant difference in PPA activity in no-blink vs. blink trials (t(23) = 5.97; p = 4.4*10-6), listed below. The sample size of our study (n = 24) is at the high end of most fMRI studies, and is in line with findings from [42], [43], who found that statistical power plateaus with N = 25 for typical fMRI studies and associated effect sizes.

After the three AB-task runs, subjects performed another six runs of the AB task under threat of mild electric shock. These data are not reported here.

#### Image acquisition

Images were collected on a General Electric 3-Tesla scanner (GE Medical Systems, Waukesha, WI) equipped with a standard clinical whole-head transmit-receive quadrature head coil. Functional images were acquired using a T2*-weighted gradient-echo, echo planar imaging (EPI) pulse sequence (33 sagittal slices, 4 mm thickness, 1 mm interslice gap; 64×64 matrix; 240 mm field of view (FOV); repetition time (TR)/echo time (TE)/Flip, 2000 ms/30 ms/60°; voxel size of 3.75×3.75×5 mm). For the localizer scan, 175 functional images were collected, while 200 functional images were collected during each attentional-blink task run. A high-resolution T1-weighted anatomical image was also acquired to assist with localization of function (T1-weighted inversion recovery fast gradient echo; 256×256 in-plane resolution; 240 mm FOV; 124×1.2 mm axial slices).

### Behavioral data analysis

For T2 performance, only T1-correct trials were analyzed. In addition, trials in which subjects indicated that they had seen the scene, but were unclear as to whether it was an indoor or outdoor scene were counted as T2-correct trials (cf. [31]). A repeated measures ANOVA was run on the behavioral-pilot data with the within-subject factors Lag (4 levels: 200, 400, 600 and 800ms) and Task (dual, single) to examine the effects of T1 processing and lag on T2 performance. Differences in T1 and T2 performance between short and long-interval trials during the mock (session 1) and MRI (session 2) sessions of the fMRI experiment were examined using paired-t tests. Analyses of the session 1 data were based on task performance during the second task run in the mock scanner only, as the first task run was considered a practice run. The design also included trials in which T2 was replaced by a scrambled image (see above). This allowed us to examine false positive rates.

### fMRI data analysis

Individual subject data were slice-time corrected, motion corrected, and analyzed in AFNI [44]. Before testing our specific hypotheses, we first wished to examine which brain areas were involved in conscious T2 perception as a replication of prior findings using our specific AB task [29], [30], [31], [45]. To this end, the functional data acquired during the AB task were analyzed using a whole-brain voxel-wise GLM with a separate regressor for each trial type, six motion estimate covariates (cf. [46]), and a second-order polynomial used to model the baseline and slow signal drift. Regressors consisted of a basis set of four TENT functions per trial type to produce separate estimated hemodynamic response functions (HRFs) for each trial type. The estimated HRFs were converted to percentage signal change values, and within-subjects contrasts between no-blink and blink trials were calculated, averaged across time points corresponding to the peak hemodynamic response during stimulus processing (4 to 8 s after stimulus stream onset), and normalized to MNI space. To normalize contrasts to MNI space, each subject's brain was first skull-stripped and warped to the MNI brain template. An average study-specific MNI brain was then created to which each subject's skull-stripped brain was warped. These warping parameters were used next to warp contrasts to the study-specific MNI template. Contrasts were subsequently smoothed using a 5-mm full-width at half-maximum Gaussian filter. These smoothed contrast maps were entered into a random-effects GLM with subject as a random factor. All statistical maps were thresholded with control for multiple comparisons using a False Discovery Rate of 0.05 (FDR; [47]). This statistical procedure guards against false activations when performing multiple hypothesis tests by controlling the fraction of false (null-hypothesis) rejections made out of the total number of rejections performed. This whole-brain voxel-wise GLM allowed us to isolate brain areas that were more strongly activated in no-blink versus blink trials. As it is unclear whether trials in which subjects perceived the layout of the scene, but were unsure whether it was an outdoor or indoor scene, would evoke similar neural activation as trials in which T2 was correctly identified as outdoor or indoor, these trials were excluded from the fMRI data analysis.

We isolated the EBA and PPA regions of each subject using the localizer task data by contrasting the brain activity in blocked presentations of bodies and scenes. Specifically, a GLM with a separate regressor for each trial type (body, natural scene), six motion estimate covariates (cf. [46]), and a second-order polynomial used to model the baseline and slow signal drift was run for each subject separately. Within-subjects contrasts between the bodies and natural scenes conditions were then calculated to identify the EBA and PPA brain regions. The isolated EBA and PPAs were composed of the 8 most active contiguous voxels in the thus identified functional clusters (p<0.0001) and collapsed across the left and right hemispheres. We also tested the selectivity of the thus identified EBA and PPA brain regions for body and scene images, respectively. To this end, for each subject, the slice-time and motion-corrected time course of activation during the localizer task was extracted for each region of interest separately. Activation values were then averaged separately for each condition (fixation, scenes, bodies), subject, and brain area (EBA, PPA) across corresponding TRs plus 2 to account for the sluggish nature of the BOLD response. Thus obtained mean activation values were then converted to percent signal change (e.g., ((bodies-fixation)/fixation)*100) and contrasted using paired t-tests to determine selectivity of EBA for bodies and PPA for scenes.

Regardless of whether the amount of resources devoted to T1 processing directly affects attentional T2 processing, it is clear that some aspect of having to encode a first target impairs the detection of a subsequently presented second target. We therefore first examined to what extent T1 processing at the level of object representation was predictive of successful T2 detection using ROI analyses. To this end, for each subject and condition (blink, no-blink) separately, the percent signal change HRFs estimated using the whole-brain voxel-wise analysis described above were extracted for the EBA and PPA regions separately. These values were contrasted using paired-t tests (*p*<.05). In addition, for each subject, we calculated the mean difference in EBA activity between blink and no-blink trials. These values were then entered in a correlation analysis examining whether individual differences in attentional processing of T1 in blink vs. no-blink trials (as indexed by EBA activity in blink vs. no-blink trials) are predictive of AB task performance.

We next investigated whether there is a reciprocal relationship between the amount of attentional resources devoted to T1 and T2 processing [11]. To this end, we examined whether activity in the PPA co-varied with activity in the EBA on a trial-by-trial basis as a function of behavior (blink, no-blink) using a psychophysiological interaction (PPI) analysis. PPI represents a measure of context-dependent connectivity, explaining regionally specific responses in one brain area in terms of the interaction between responses in another brain region and a cognitive or sensory process [37], [38]. If T1 and T2 directly share limited resources one would expect differential coupling of EBA and PPA activity in blink vs. no-blink trials. To examine this, for each subject, we reran the GLM described above, but with the insertion of three additional regressors. The first regressor, the physiological variable, was the detrended time series of the EBA (averaged across the 8 voxels of the left and the right EBA ROIs). The second and third regressors, or psychophysiological interaction terms, were created by calculating the product of the detrended EBA activation time-course after deconvolution with a gamma function (cf. [48]) and the vector of the psychological variable of interest (a vector containing 1's for TRs during which the trial type of interest (blink or no-blink) occurred, and 0's for all other TRs). To determine whether activation in the PPA was differentially predicted by two psychophysiological interaction terms, the parameter estimate for each interaction term was converted to a Z score through Fisher transformation, and collapsed across all voxels in the PPA ROI for each subject separately. These individual normalized Z scores values for blink and no-blink trials were then contrasted in SPSS using a paired *t*-test, yielding connectivity patterns with the PPA ROI (thresholded at *p*<0.05).

## Results

### Behavioral pilot experiment

**Figure 2:**
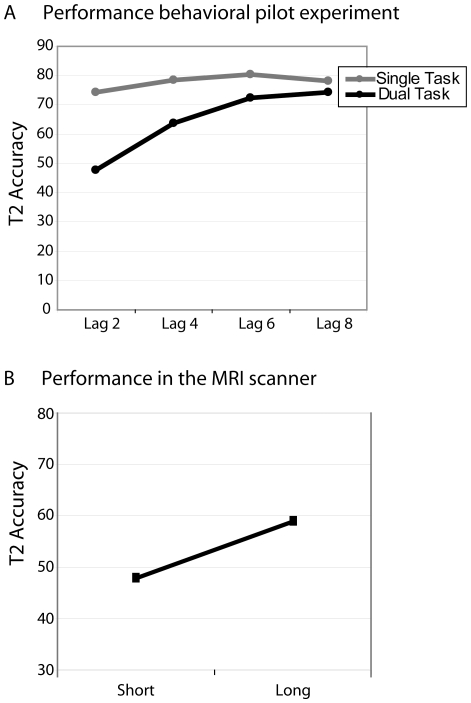
Behavioral T2 performance. A: Data from the behavioral pilot experiment. T2 accuracy as a function of Lag (200, 400, 600 or 800 ms) and Task Condition (single, dual). B: Behavioral data collected in the MRI scanner. T2 accuracy as a function of Lag (short, long).

As expected, and can be seen in Figure 2A, the behavioral pilot experiment established that T2-scene detection was substantially lower when subjects were required to detect both T1 and T2 (dual task) versus when they only had to detect T2 (single task), as reflected by a main effect of condition (F(1,15) = 23.2; p<.001). In particular, T2 detection rates were lower in the dual vs. single task when T2 followed T1 relatively quickly (interaction between condition and Lag; F(3,45) = 3.3; p = .05). In addition, in the dual-task condition, T2 performance was substantially lower when T2 followed T1 within the time window of the attentional blink, as reflected by a significant main effect of Lag (F(1,15) = 5.0; p = .016). These results are trade-mark features of the AB [5], [7], and illustrate that an attentional blink can be obtained with this paradigm. In both the single- and dual-task conditions, the average percentage of trials in which participants perceived the layout of the scene, but were unsure whether is was an outdoor or indoor scene was relatively low (8.3% and 9.4% respectively).

### fMRI experiment

#### Behavioral results: Session 1

As expected, subjects showed an AB, detecting both targets in a significantly smaller portion of short-interval compared to long-interval T2-present trials (t(23) = 2.1, one-tailed p = .022; 52.4% vs. 61.3%). Mean T1 accuracy was 84.1% and not affected by the temporal distance between the two targets (t(23) = .71, p = .48; 85.3% in short-interval vs. 82.4% in long-interval T2-present trials). The mean T2 false alarm rate was 7% and significantly below the T2 detection rates in short-interval T2 present trials (t(23) = 10.7, p<.001). This latter finding established that T2 false alarm rates were negligible compared to true T2 detection rates. The mean percentage of short- and long-interval trials in which participants perceived the layout of the scene, but were unsure whether it was an outdoor or indoor scene were, respectively, 9.5% and 9.0%.

#### Behavioral results: Session 2

In the MRI scanner, subjects also detected both targets in a smaller portion of short-interval compared to long-interval T2-present trials (t(23) = 1.8; one-tailed p = .046; 47.9% vs. 58.8%), evidencing an AB (see Figure 2B). This was true regardless of whether trials in which participants perceived the layout of the scene, but were unsure whether it was an outdoor or indoor scene (i.e., unknown-scene trials) were counted as T2-correct trials (t(23) = 1.7; one tailed p = .049). The mean percentage of short- and long-interval unknown-scene trials were, respectively, 6.9% and 9.6%. Mean T1 accuracy was 86.4% and was somewhat higher in short-interval (89.2%) compared to long-interval (83.6%) trials (t(23) = 2.8; p = .011).

One surprising aspect of the present findings was the lower percentage of no-blink trials in both the short and the long T1-T2 interval condition in sessions 1 and 2 of the fMRI experiment compared to the behavioral pilot experiment. One possible explanation for these differences in T2 detection rates may be the fact that the fMRI experiment only included subjects who detected both targets on at least 30%, but on no more than 70% of short-interval trials. Although a reanalysis of the behavioral pilot study data including only subjects who met this criterion (n = 10) showed somewhat lower T2 detection rates in general for this subgroup, T2 accuracy rates were still substantially higher for both interval conditions (short interval: 60%, long interval: 72%) compared to those observed for the fMRI experiment (sessions 1 and 2). As the two experiments used different groups of subjects and sample sizes were relatively small, another possibility is that the differences in T2 detection rates between experiments can be explained by the large variability in AB task performance, which is typically observed between individuals (e.g., [49]). A final possibility is that presenting the stimuli through the goggles in the fMRI experiment generally increased the perceptual difficulty of the AB task. This possibility is supported by the somewhat higher mean T1 accuracy rate (91% averaged across conditions) obtained in the behavioral pilot experiment. Regardless of what may account for the discrepancy in findings between experiments, of greatest importance is that even though T2 accuracy rates were lower in the fMRI experiment, this was equally true for both the short and long T1-T2 interval conditions.

#### fMRI results (voxel-wise analysis): Brain areas associated with conscious T2 perception

**Figure 3:**
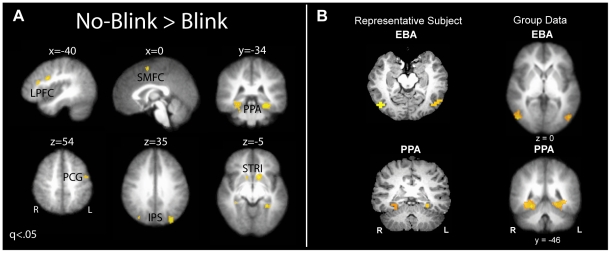
Brain regions associated with conscious T2 perception (A) and body parts and natural scenes (B). A: Frontoparietal network associated with conscious T2 perception (*p*<0.05 after controlling for False Discovery Rate). LPFC, lateral prefrontal cortex; SMFC, superior medial frontal cortex; PCG, precentral gyrus; IPS, intraparietal sulcus; PPA, parahippocampal place area; STRI, striatum. B: Localizer task data. The ‘representative subject’ map shows the 8 most active contiguous voxels for each region of interest (p<.0001). The ‘group data’ map is thresholded at q<.05 (or p<.0006).

**Figure 4:**
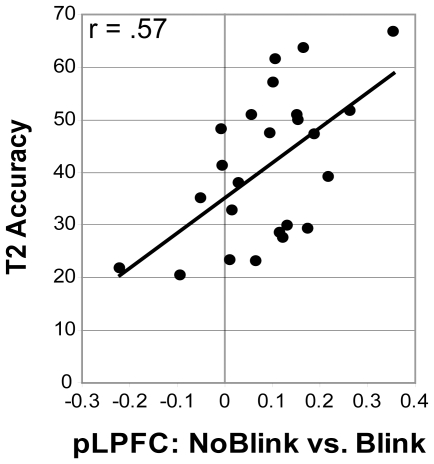
The relationship between individual differences in prefrontal brain activity and T2 accuracy. Greater activity in left posterior lateral prefrontal cortex selectively predicted higher T2 detection rates.

Before testing our specific hypotheses, we first wished to examine which brain areas were involved in conscious T2 perception as a replication of prior findings using our specific AB task [29], [30], [31], [45]. Greater activity was observed in a network of frontal and parietal brain areas in no-blink vs. blink short-interval trials (Table 1, Figure 3a). This network included left lateral prefrontal cortex, superior medial frontal cortex, and bilateral parietal cortex and is similar to the frontoparietal network of brain areas previously implicated in the AB [29], [30], [31], [45]. A regression analysis with T2 accuracy as a predictor of T2-detection-related activity in this network revealed that greater activity in left posterior lateral prefrontal cortex (LPFC, see Figure 3a) in no-blink vs. blink trials was associated with higher T2 accuracy rates (r = .57, p = .003, n = 24; see Figure 4). None of the other frontal and parietal brain areas involved in conscious T2 perception showed this relationship between activity and performance. In addition, further replicating findings from studies using similar task stimuli [31], conscious scene (T2) perception was associated with greater activity in the PPA (see Figure 3a). This latter finding is in line with previous observations that attention to T2 modulates activity in lower-level T2 representation areas [28], [31], [32], [34], [35], [36].

**Table 1:** Brain areas involved in conscious T2 perception.

Brain Region	Hemisphere	MNI Coordinates			Max Intensity
		x	y	z	
pLPFC	left	−40	7	29	5.8
aLPFC	left	−42	28	19	5.1
PCG	left	−55	−9	54	5.0
SMFC	bilateral	0	−3	67	5.8
IPS	left	−32	−86	33	6.6
	right	34	−82	37	4.8
PPA	left	−30	−38	−21	6.8
	right	32	−30	−30	6.5
POF	left	−16	−60	10	4.8
	right	22	−61	14	6.5
ITG	left	−51	−46	−31	4.5
Cereb	right	36	−54	−44	5.8
Striatum	left	−12	17	−6	6.1
	right	12	6	−4	5.6
	right	12	15	−11	4.7
Sub Nigra	left	−12	−16	−18	4.5

MNI coordinates (xyz) and t-values are listed for brain regions showing greater activity in no-blink compared to blink trials (*p*<0.05 after controlling for False Discovery Rate). Abbreviations: L, left; R, right; pLPFC, posterior lateral prefrontal cortex; aLPFC, anterior lateral prefrontal cortex; PCG, precentral gyrus; SMFC, superior medial frontal cortex; IPS, intraparietal sulcus; PPA, parahippocampal place area; POF, parietooccipital fissure; ITG, inferotemporal gyrus; Cereb, cerebellum, Sub Nigra, substantia Nigra.

#### fMRI results (ROI analyses): Effects of T1 encoding on T2 detection rates

**Figure 5:**
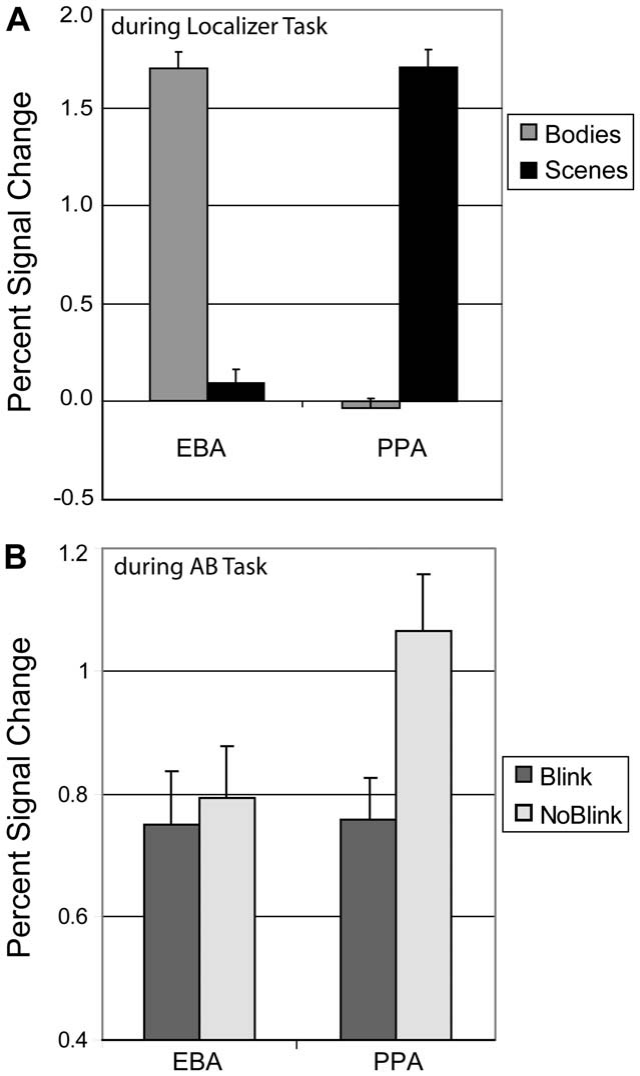
T1- and T2-object specific activity during the localizer task (A) and the AB task (B). A: Selective activity in the EBA and PPA during the localizer task to bodies and scenes, respectively. B: Activity in the EBA and PPA during the AB task as a function of conscious T2 perception (blink, no-blink). While the EBA was equally active in no-blink and blink trials, the PPA exhibited significantly greater activation when T2 was consciously perceived.

The ROI analysis confirmed selectivity for bodies in the EBA and for scenes in the PPA (see Figures 3b and 5a). During the localizer scan, as expected, bodies elicited significantly greater activation in the EBA than scenes (t(23) = 18.3; p<.001) and compared to fixation (t(23) = 20.1; p<.001), while scenes elicited significantly greater activation in the PPA than bodies (t(23) = 18.0; p<.001) and compared to fixation (t(23) = 18.9; p<.001). Of further importance, the EBA did not show greater activation during scene presentation compared to fixation (t(23) = 1.4; p = .18), while the PPA did not show greater activation during body presentation compared to fixation (t(23) = 0.7; p = .50). The mean MNI coordinates of the isolated EBA and PPI ROIs (x,y,z: −50,−68,0 (left EBA); 56,−78,−2 (right EBA); −26,−50,12 (left PPA); 26,−44,−12 (right PPA)) are consistent with the known location of the EBA and PPA (see Figure 3b; [39], [40]).

**Figure 6:**
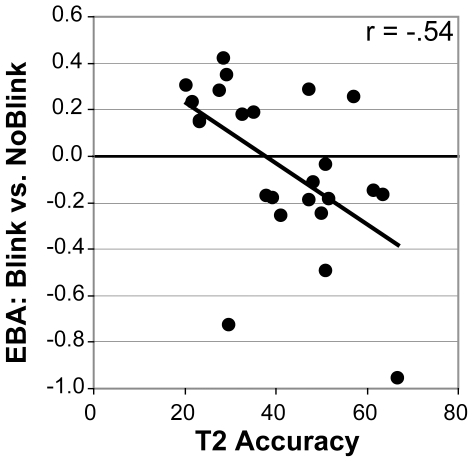
T1-related EBA activity predicts T2 accuracy across participants.

We next examined to what extent T1 processing at the level of object representation was predictive of successful T2 detection using individual-subject ROI analyses. Across subjects, mean activity in the EBA did not vary as a function of conscious T2 perception: The EBA was equally active in blink and in no-blink trials (Figure 5b; t(23) = .97; p = .34). We then examined whether EBA activity in blink compared to no-blink trials was a significant predictor of individual differences in T2 accuracy. Indeed, and in line with the idea that T1 encoding can significantly affect T2 detection, we found that those subjects who activated the EBA more strongly in blink vs. no-blink trials generally showed a bigger blink effect (Figure 6; r = −.54, n = 24, p = .006). No such relationship was observed for the PPA (r = −.22, n = 24, p = .30), although this area was generally more strongly activated by consciously identified T2s (Figure 5b; t(23) = 5.97; p = 4.4*10-6), in line with the whole-brain voxel-wise analysis results reported above. To summarize, although the EBA was generally not more strongly activated in blink trials, activity in this region in blink vs. no-blink trials selectively predicted an individual's ability to detect both targets successfully.

Activity in the EBA likely reflects a combination of bottom-up, T1-driven processing and top-down attentional modulation. It is thus possible that differences in bottom-up T1 processing in blink vs. no-blink trials cancelled out differences in top-down attentional T1 processing in blink vs. no-blink trials and can explain our null finding of no T2 detection-related differences in mean EBA activity. This possibility seems unlikely, however, as studies using the high temporal resolution of ERPs have not found any differences in early, sensory T1 processing as a function of T2 detection [23], [25], [50], [51], [52]. Nevertheless, to examine this possibility more directly for the current data set, in a post-hoc analysis, we used T1 difficulty (or average T1 accuracy) as an index of bottom-up processing, and compared EBA responses to “easy” T1's (the five body stimuli with the highest accuracy rates; average T1 accuracy: 93.5%) and “hard” T1's (the five body stimuli with the lowest accuracy rates; average T1 accuracy: 84.8%). Although the difference in T1 accuracy between easy and hard T1's was highly significant (t(23) = 4.6, p<.0001), no differences in average EBA peak percent signal change were observed as a function of T1 difficulty (p = .62). This finding argues against the possibility that differences in bottom-up T1-related processing obscured possible differences in top-down attentional processing of T1. The observed cross-subject relationship between T1 encoding-related processes in EBA and AB size therefore likely reflects individual differences in top-down processing strategies rather than in bottom-up T1 processing. This conclusion receives additional support from a further correlation analysis showing no relationship across subjects between average T1 accuracy and average EBA activity in either blink (p = .69) or no-blink (p = .86) trials.

#### fMRI results (PPI analysis): Resource sharing and the AB

We next tested the hypothesis that T1 and T2 share limited attentional resources [11]. To this end, we examined - within subjects - whether activity in PPA co-varied with activity in EBA on a trial-by-trial basis as a function of behavior (blink, no-blink) using a PPI analysis. PPI represents a measure of context-dependent connectivity, and enables the identification of changes in the functional coupling between two brain areas depending on the psychological context [37], [38]. The PPI analysis revealed no differences in coupling of activity between the EBA and PPA during blink compared to no-blink trials (t(23) = .19; p = .85). Thus, this analysis provided no evidence for the resource-sharing account of the AB which assumes a reciprocal relationship between T1 and T2 attentional processing. One possibility is that the observed large individual variability in T1 encoding in blink vs. no-blink trials obscured this relationship (see Figure 6). We therefore examined post-hoc whether individual variability in EBA activity in blink vs. no-blink trials predicted individual differences in the coupling of activity between EBA and PPA in blink vs. no-blink trials. To this end, we ran a regression analyses with the peak percent signal change difference in EBA activity between blink and no-blink trials as a predictor of the normalized Z scores of the psychophysiological interaction effect obtained for PPA (see above). No significant relationship was observed in this analysis.

## Discussion

The present study used event-related fMRI to investigate the neural mechanisms underlying a major limitation in human information processing: The ability of the brain to process two temporally-close meaningful stimuli. By using correlates of processing in sensory cortices specifically related to either T1 selection or T2 selection and functional connectivity analyses, we examined how attentional processing of T1 may determine the AB to T2. First, we found that attentional processing of T1 at the level of the visual cortex predicted T2 detection rates: Those individuals who activated the T1 encoding area (i.e., the EBA) more strongly in blink versus no-blink trials generally exhibited a bigger AB. This finding is in line with the general notion that the AB arises from attentional demands of T1 for selection. In addition, by revealing a relationship between T1-related activity in visual cortex and the AB, this observation extends previous findings showing that T2-related activity in early visual areas is modulated during the AB [28], [32], [36]. Second, we found that the coupling of activity between T1 and T2 encoding areas (i.e., the EBA and PPA) did not vary as a function of conscious T2 perception. Although one should be cautious in interpreting any null finding, this result provides no evidence for a reciprocal relationship between the amount of attentional resources devoted to T1 and T2 processing, as the resource-sharing account of the AB predicts [11]. Below, we discuss these observations and their implications in more detail.

In the current study, we found that those individuals who activated the EBA more strongly in blink vs. no-blink trials, generally detected T2 on a smaller percentage of trials. This cross-subject relationship between attentional processing of T1 and the size of the AB is consistent with the general notion that the AB arises from attentional demands of T1 for selection (for recent reviews, see [14], [15]), and indicates that these demands are reflected at the level of visual cortex. Previous AB studies have shown reliable modulation of T2 processing in temporal, frontal, and parietal brain regions, as well as visual regions, including the primary visual cortex [28], [29], [30], [31], [32], [34], [36]. This has lead to the proposal that the AB arises because T1 processing prevents the iterative feedback process between higher cortical (e.g., parietal, temporal) areas and lower visual areas required for T2 identification from being completed before masking of T2 by the items presented immediately after it [36], [53]. Extending these findings, the current data suggest that the AB may also be reflected in the strength of the iterative feedback process required for T1 identification, as indexed by EBA activity. More generally, these findings support the notion that competition for attentional resources not only occurs when stimuli are presented simultaneously, but also when they are presented separately in close temporal proximity.

The current data are less readily explained by models of the AB that postulate that the AB is due to mechanisms that are independent of, and/or subsequent to, T1 processing (e.g., [8], [15]). Notably, however, in a recently formulated theory of the AB, the boost and bounce theory [15], a critical role is proposed for feedback connections between higher order brain areas and visual areas in the AB. According to this theory, the AB is not due to T1 processing per se, but due to feedback responses elicited by the first distracter presented after T1. Specifically, the boost and bounce theory proposes that T1 elicits transient excitatory feedback activity meant to provide access to working memory. However, since it takes approximately 100 ms for this excitatory feedback to arrive in visual areas, accidentally, the post-T1 distracter is also boosted. This results in a subsequent strong inhibitory feedback response, which, in effect, closes the gate to working memory for T2. Importantly, in this model, the strength of this inhibitory response depends on the strength of the excitatory response elicited by T1. An alternative possibility is thus that the observed relationship between T1 processing in EBA and the size of the attentional blink can be explained by a third factor, namely differences in the strength of the inhibitory response elicited by the post-T1 distracter, rather than by differences in T1 attentional selection demands per se. Unfortunately, our data cannot distinguish post-T1 distracter processing from other stimulus processing and hence, do not dissociate between distracter- and target-based models of the AB. Future neuroimaging studies are necessary to delineate whether and how the post-T1 distracter might affect conscious T2 perception.

Although a relationship was observed - across subjects - between EBA activity in blink vs. no-blink trials and AB size, mean EBA activity did not differ significantly between blink and no-blink trials. It is possible that large individual differences in task processing strategies can account for this null finding. Indeed, recent behavioral studies have shown that the attentional settings of the observer can greatly influence the temporal dynamics of attention [54], [55], [56]. The observed relationship between T2 detection-related EBA activity and AB size in the current study may reflect such individual differences in attentional settings. As EBA activity did not vary as a function of T1 difficulty, it is conceivable that this relationship reflects inter-individual differences in top-down attentional allocation rather than in bottom-up T1-driven processing. A key challenge for (computational) models of the AB is to explain how individual differences in top-down attentional settings interact with the AB phenomenon (see [13]).

Further to examining the relationship between attentional processing of T1 at the level of the visual cortex and the AB, the current study investigated whether there is a reciprocal relationship between the amount of attentional resources devoted to T1 and T2 processing using a PPI analysis. According to the resource-sharing account of the AB, T1 and T2 are processed in parallel and directly compete for shared limited resources [11]. In contrast to serial-stage models of the AB, in which the AB is an all-or-none phenomenon, this account predicts a reciprocal relationship between the amount of attentional resources devoted to T1 and T2 processing. A PPI analysis of our fMRI data found no differential coupling in activity between the EBA and PPA as a function of T2 detection, arguing against the idea of resource sharing. Of course, one should always be cautious in interpreting a null finding, and we cannot fully exclude the possibility that this null finding is attributable to other factors, such as the relatively small AB observed, noise in the data, or the required task switch between T1 and T2, which may have introduced an additional bottleneck in the processing stream [57]. It should be noted, however, that, while one ERP study observed a reciprocal relationship between the amount of resources, as indexed by the amplitude of the P3b, devoted to T1 and T2 processing [20], other ERP studies did not observe such a relationship [25], [26]. Future neuroimaging studies are necessary to further examine the idea of resource sharing at the neural level, and to replicate our null finding.

Previous neuroimaging studies have generally implicated higher-order frontal and parietal brain areas in the AB [29], [30], [31], [45]. In the current study, the posterior lateral prefrontal cortex selectively predicted AB task performance, suggesting that this area in particular may play an important role in the AB. It is notable in this respect that several fMRI studies have identified the posterior lateral prefrontal cortex as a core brain area underlying a central bottleneck of information processing that severely limits our ability to multitask [58], [59]. For example, using time-resolved fMRI, Dux et al. [58] found that this brain area exhibited serial queuing of response selection activity under dual-task conditions. The posterior lateral prefrontal cortex may thus play a critical role in successful performance on dual tasks, such as the AB task.

To conclude, the current data are in line with the idea that the AB arises from attentional demands of T1 for selection and highlight the importance of individual differences in attentional strategies in explaining AB task performance.
